# Effects of Salicornia-Based Skin Cream Application on Healthy Humans’ Experimental Model of Pain and Itching

**DOI:** 10.3390/ph15020150

**Published:** 2022-01-26

**Authors:** Rocco Giordano, Giulia Erica Aliotta, Anja Stokholm Johannesen, Dina Voetmann-Jensen, Frederikke Hillebrand Laustsen, Lasse Allermann Andersen, Aman Rezai, Malthe Fredsgaard, Silvia Lo Vecchio, Lars Arendt-Nielsen, Mette Hedegaard Thomsen, Allan Stensballe

**Affiliations:** 1 Center for Neuroplasticity and Pain (CNAP), SMI, Department of Health Science and Technology, Faculty of Medicine, Aalborg University, 9220 Aalborg, Denmark; rg@hst.aau.dk (R.G.); gea@hst.aau.dk (G.E.A.); slv@hst.aau.dk (S.L.V.); lan@hst.aau.dk (L.A.-N.); 2 Translational Biomarkers in Pain and Precision Medicine, Department of Health Science and Technology, Faculty of Medicine, Aalborg University, 9220 Aalborg, Denmark; asjo17@student.aau.dk (A.S.J.); dvoetm17@student.aau.dk (D.V.-J.); flaust17@student.aau.dk (F.H.L.); laande17@student.aau.dk (L.A.A.); mrezai15@student.aau.dk (A.R.); 3 Department of Energy Technology, Aalborg University, 6700 Esbjerg, Denmark; mfre@energy.aau.dk (M.F.); mht@energy.aau.dk (M.H.T.); 4 Department of Medical Gastroenterology, Mech-Sense, Aalborg University Hospital, 9000 Aalborg, Denmark

**Keywords:** Salicornia, itch, pain, human experimental model, neurogenic inflammation

## Abstract

Halophyte plants are salt-tolerant and are acclimated for growth in saline soils such as along coastal areas. Among the halophytes, the Salicornia species have been used as both folk medicine and functional food for many years due to their high levels of bioactive compounds with supposed anti-inflammatory and antioxidative effects. However, the properties of Salicornia bioactive extracts on pain and itching still remain unclear. In this study, 30 healthy volunteers were randomized to treatments with 10% Salicornia-based cream or placebo cream for 24 or 48 h. On day 0, and 24 or 48 h post cream application, cold/heat detection and pain thresholds, mechanical pain thresholds and sensitivity, trans-epidermal water loss, histamine- and cowhage-evoked itch, and micro-vascular reactivity (neurogenic inflammation) were assessed to evaluate the analgesic, anti-pruritogenic and vasomotor effects. Skin permeability was reduced in the Salicornia-treated area for 48 h compared with 24 h application (*p*-value < 0.05). After 48 h of application, a decrease in mechanical-evoked itching (hyperkinesis) compared with 24 h treatment (*p*-value < 0.05) and increased warm detection and heat pain thresholds (*p*-value < 0.05) was found. Histamine-induced neurogenic inflammation showed a significant reduction in the cream-treated areas after 48 h compared with 24 h (*p*-value < 0.05). The results of this study indicate the overall inhibitory effect of Salicornia on hyperkinesis (mechanically evoked itch), the analgesic effect on thermal sensation, and modulation of the skin barrier architecture. Further studies are needed for the assessment of the long-term effects.

## 1. Introduction

Medical plants have been used in folk medicine for thousands of years due to their high load capacity of active molecules. In this direction, there is a continued interest in the possibility of using these plant species to discover new therapies and medications through the characterization of its bioactive components and active secondary metabolites [1]. Halophyte farming in synergy with aquaculture may be used to create high-value-added products in the cosmetic and pharmaceutical industry through the implementation of continual use of resources and waste through prevention, reduction, and recycling. Halophytes are salt-tolerant plant species that grow in salt marshes near costs and have adapted to and developing salt tolerance (200 mM NaCl), resistance to high temperatures, as well as light intensity [1]. Halophytes include several species even though they do not form a systematical group, and, phylogenetically, they are not related to each other.

*Salicornia ramosissima* (*S. ramosissima)* is a part of the Chenopodiaceae family and grows in salt marshes and on coastal areas of Europe, the Iberian Peninsula, and Northwest Africa [2,3]. This species shows anti-inflammatory and antioxidative effects due to the presence and action of secondary metabolites, such as flavonoids and phenolic compounds [4]. The active compounds have proven efficacy in a study showing that the antioxidative and photoprotective abilities of ethyl acetate fraction of *S. ramosissima* were higher than available antioxidants [5]. In addition, UV protection was found to be more efficient than commercial UV-filters because of its higher production of a large variety of phytochemicals contributing to the protective effects of the extract [5]. *S. ramosissima* is one of the least characterized plants of the halophyte species, but it is included in the species aggregate *Salicornia europaea* (S. *europaea*), which has been studied to a greater extent for the past years [6]. Due to the morphological and phenotypical similarities between *S. europaea* and *S. ramosissima*, it is difficult to differentiate between halophytes’ two typologies. Therefore, it is assumed that the plants have very close similarities in their expression of secondary metabolites, which regulate their anti-inflammatory and antioxidant effects [6,7]. It has been demonstrated that Salicornia possesses anti-inflammatory opportunities, as its extracts are capable of inhibiting proinflammatory cytokines, such as TNF-α, IL-1β, and IL-6, as well as oxidative stress markers such as HO-1 in mice models of Parkinson’s disease [8]. In addition, the antioxidative effect of Salicornia and other six halophytes were investigated by an evaluation of 2,2-diphenyl-1-picrylhydrazyl radical scavenging, demonstrating that the extract possesses great anti-inflammatory activity by inhibiting the nitrogen-oxide production [9], hence the interest in new safe therapies for skin diseases as many involve pain, itching, and inflammation [10,11]. The effects of Salicornia extract on skin conditions have recently been evaluated in a study that looked into the effect of the prolonged application of aqueous extracts from *S. europaea* in a skin sunburn model in women [12]. The aims of this placebo-controlled study were to investigate the analgesic and antipruritic effects, and effects on neurogenic inflammation at 24 and 48 h after topical application of *S. ramossisma* in healthy subjects.

## 2. Results

### 2.1. Demographics for Evaluated Cohort

Thirty subjects were recruited for the study and divided (Table 1) into two groups based on the duration of treatment with *S. ramosissima* Cream (SC) and Vehicle Cream (VC). Group 1 received the treatments for 24 h, whereas group 2 received treatments for 48 h. All participants tolerated the prolonged administration of SC and completed the procedures, as no safety issues occurred during or after the study.

### 2.2. Trans-Epidermal Water Loss for Assessments of Skin Irritation and Tissue Permeability

**Figure 1:**
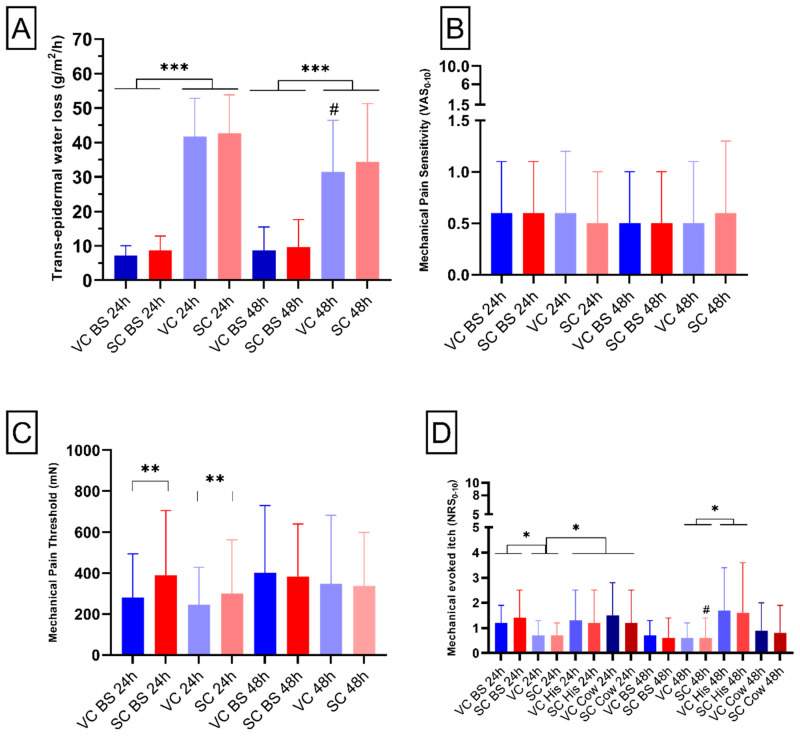
Trans-epidermal water loss, mechanical pain, and itch assessments. (**A**) Bar chart reports quantification of alteration of skin barrier measured in g/m^2^/h. (**B**) Bar chart depicts pain sensitivity reported by the participants on a numerical rating scale (NRS) from 0 to 10. (**C**) Bar chart shows mechanical pain thresholds reported by the subjects. (**D**) Bar chart shows itching reported on a NRS (0–10) by the subjects. Blue colors represent areas treated with vehicle cream (VC), whereas red colors indicate areas treated with S. ramosissma-based cream (SC). “BS” indicates data obtained at baseline. The label “24 h” reports data relative to group 1 and “48 h” indicates data relative to group 2. “His” and “Cow” indicates data relative to area treated with histamine or cowhage, respectively. Mean and standard deviation of the mean are depicted. *, **, ***, = RM-ANOVA test significance. # = *t*-test statistical significance.

In order to evaluate the effects of Salicornia and vehicle cream application on the homeostasis and the permeability of the skin, trans-epidermal water loss (TEWL) was evaluated [13]. The analysis of TEWL showed significant higher values compared with baseline for subjects of both groups (mean effect of time: 24 h application: F_1,14_ = 202.8, *p*-value < 0.001; 48 h application: F_1,14_ = 23.2, *p*-value < 0.001, Figure 1A). This underlines that both SC and VC treatment induced an increase in TEWL after 24 and 48 h of application. Moreover, in both groups no effect was reported for treatment (24 h: *p*-value = 0.47; 48 h: *p*-value = 0.31, Figure 1A), indicating no difference of effect between vehicle and *S. ramosissima* cream. The treatment × time interaction was also not significant (24 h: *p*-value = 0.9; 48 h: *p*-value = 0.53, Figure 1A). A significant decrease in TEWL was found when group 1 and group 2 were compared in the vehicle-cream-treated area (*p*-value < 0.05, Figure 1A).

### 2.3. Mechanical Pain Assessments

With the aim of evaluating pinprick hypoalgesia, defined as reduced pain in response to a normally painful stimulus, induced by the Salicornia or vehicle cream, mechanical pain sensitivity (MPS) and mechanical pain threshold (MPT) were performed [14,15]. For the analysis of the mechanical pain sensitivity (MPS), we did not show any effect of time (24 h: *p*-value = 0.76; 48 h: *p*-value = 0.4, Figure 1B), treatment (24 h: *p*-value = 0.21; 48 h: *p*-value = 0.85, Figure 1B) or treatment  ×  time interaction (24 h: *p*-value = 0.83; 48 h: *p*-value = 0.15, Figure 1B), demonstrating that application of SC after 24 or 48 h does not affect the touch perception of healthy subjects.

The analysis of the mechanical pin prick pain threshold (MPT) after 24 h application showed a significant effect of treatment (F_1,14_ = 1.1, *p*-value < 0.01, Figure 1C), probably underlying the difference between vehicle and *S. ramosissima* cream both at baseline and after 24 h application. No effect of treatment after 48 h application was found (*p*-value = 0.8, Figure 1C). Furthermore, no effect of time (24 h: *p*-value = 0.31; 48 h *p*-value = 0.2, Figure 1C) nor the treatment × time interaction (24 h: *p*-value = 0.37; 48 h *p*-value = 0.9, Figure 1C) was found for both application times.

### 2.4. Mechanically Evoked Itch

The aim of the mechanical-evoked itch (MEI) assessment was the evaluation of enhanced itching to normally itch-provoking stimuli or reduced itching threshold in response to a stimulus [16]. The analysis of MEI after 24 h application showed only a significant effect of time (F_2,28_ = 4.6; *p*-value < 0.05, Figure 1D), with a statistical difference between baseline vs. 24 h (*p*-value < 0.05) and between 24 h vs. post pruritogens application (*p*-value < 0.05). No other main effect or interaction had significant results. 

The analysis of MEI after 48 h application showed a significant interaction between time × pruritogen (F_1,14_ = 6.9; *p*-value < 0.05, Figure 1D), indicating, as expected, an increase in MEI after histamine application regardless of treatment. As for the 24 h application, no other main effects or interactions were significantly different. A significant decrease in MEI was reported when comparing group 1 and group 2 in the Salicornia cream-treated area (*p*-value < 0.05, Figure 1D). 

### 2.5. Thermal Assessments

In order to evaluate thermal hypoalgesia and analgesia, defined as diminished pain or absence of pain in response to stimulation, induced by the Salicornia or vehicle cream, thermal assessments such as cold detection and pain threshold (CDT, CPT), warm detection threshold (WDT) heat pain threshold (HPT), and supra-threshold heat sensitivity (STHS) were performed [14,15]. The non-parametric Friedman test for CDT, for both the 24 and 48 h treatment, did not show any significant difference (24 h: chi-square = 5.20, *p*-value = 0.16; 48 h chi-square = 5.9, *p*-value = 0.12, data not shown). 

The analysis of CPT and STHS did not show any statistical main effect or interaction regardless of the application time of the creams (*p*-value > 0.05, data not shown).

**Figure 2:**
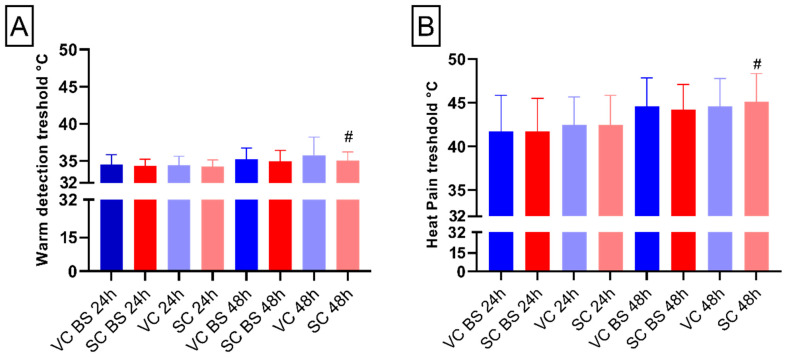
Thermal assessments. (**A**) Bar chart shows warm detection measured in °C degrees. (**B**) Bar chart reports heat pain threshold measured in °C degrees. Blue colors represent areas treated with vehicle cream (VC), whereas red colors indicate areas treated with *S. ramosissima*-based cream (SC). “BS” indicates data obtained at baseline. Error bars report standard deviation (SD). # = *t*-test statistical significance.2.6. Microvascular Reactivity (Neurogenic Inflammation).

In regard to the WDT and HPT, differences between the two groups were found with increased value after 48 h application compared with 24 h application in the area treated with Salicronia cream (WDT: *p*-value < 0.05; HPT: *p*-value < 0.05 Figure 2A,B), underlying the key effect of application time. 

**Figure 3:**
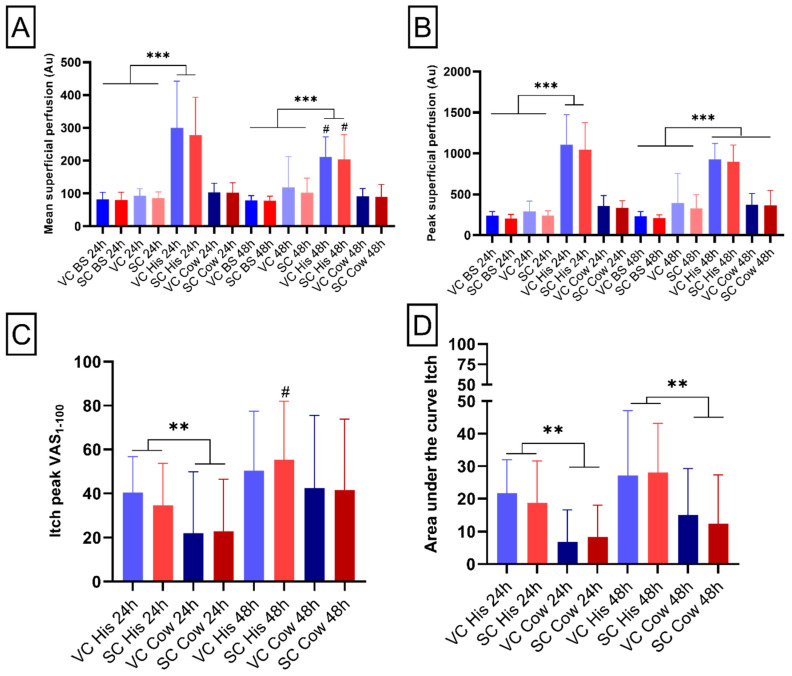
Micro-vascular reactivity and itching assessments. (**A**,**B**) Bar charts report the means and peaks of blood perfusions at baseline, after creams application and after histamine (his) and cowhage (cow) induction. (**C**,**D**) Bar chart depict peak and area under the curve pain reported by the participants on a scale from 0 to 100. Blue colors represent areas treated with vehicle cream (VC), whereas red colors indicate areas treated with *S. ramosissima*-based cream (SC). “BS” indicates data obtained at baseline. “24 h” reports data relative to group 1 and “48 h” indicates data relative to group 2. “His” and “Cow” indicates data relative to area treated with histamine or cowhage, respectively. Error bars report standard deviation (SD). **, ***, = RM-ANOVA test significance. # = *t*-test statistical significance.

One of the common cutaneous responses to pruritogens application is neurogenic inflammation or neurogenic flare, which is a brief increase in superficial blood perfusion [17]. The full-field laser perfusion imaging (FLPI) allows assessment of evoked neurogenic inflammation and evaluation of cutaneous inflammation [18]. FLPI mean values for both 24 h and 48 h application showed an interaction between time × pruritogen (24 h: F_2,28_ = 45.6, *p*-value < 0.001; 48 h: F_2,28_ = 97.0, *p*-value < 0.001, Figure 3A). An increase in the superficial blood perfusion was proven after the histamine application compared with baseline and after the VC and SC application. A significant decrease in superficial blood perfusion was proven after the histamine application between group 1 and group 2 in both the SC and VC areas (*p*-value < 0.05, Figure 3A).

The analysis of FLPI peak values for both 24 h and 48 h application showed an interaction between time × pruritogen (24 h: F_2,28_ = 69.2, *p*-value < 0.001; 48 h: F_2,28_ = 100.6, *p*-value < 0.001, Figure 3B. An increase in the superficial blood perfusion after the histamine application was proven in groups 1 and 2, compared with both the baseline and after the SC and VC application. In addition, an increase was also indicated after the cowhage application in group 2 when compared with the baseline values.

### 2.6. Pruritonergic Itching and Pain Intensities

The analysis of peak itching values for 24 h application showed a main effect of itching (F_1,14_ = 11.6, *p*-value < 0.01). An increased itch peak was proven after histamine application compared with cowhage application (*p*-value < 0.01, Figure 3C). No statistical differences were present for the application at 48 h in regard to the pruritogens. In addition, a difference between groups was present regarding histamine application, showing an increased itch peak in the area treated with Salicornia cream after 48 h compared with the application at 24 h (*p*-value < 0.05, Figure 3C).

Additionally, the area under the curve (AUC) analysis of itching after applications at 24 h and 48 h showed a main effect of itching (24 h: F_1,14_ = 29; 48 h: F_1,14_ = 9.49; *p*-value < 0.01) with an increased itch peak after histamine application compared with cowhage application (*p*-value < 0.01, Figure 3D).

The analysis of peak pain values for both the 24 h and 48 h application did not show any significant differences after application of either pruritogen between the areas treated with vehicle or Salicornia cream (24 h: chi-square = 3.3, *p*-value = 0.34; 48 h chi-square = 1.81, *p*-value = 0.61, data not shown). Moreover, similar results were reported for the analysis of the AUC for both the 24 h and 48 h application (24 h: chi-square = 4.4, *p*-value = 0.22; 48 h chi-square = 2.24, *p*-value = 0.5, data not shown).

**Figure 4:**
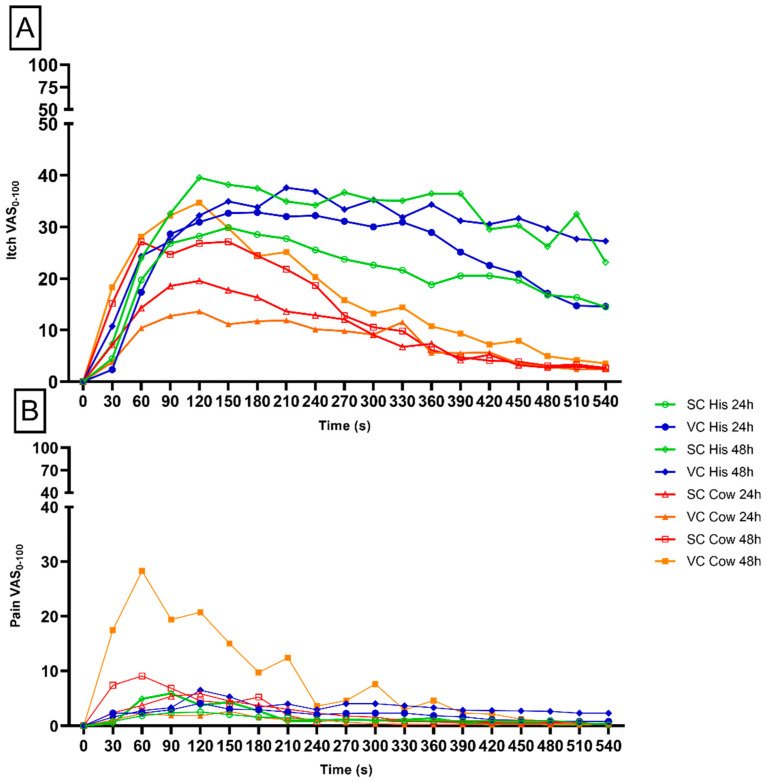
Itching and pain temporal profile. The green and red lines depict areas treated with *S. ramosissima* cream, whereas blue and orange indicate areas treated with vehicle cream. Intensities (VAS) for itching (**A**) and pain (**B**) were reported on a scale from 0 to 100 (*n* = 30). Time of assessment for a total of 9 min is reported in seconds (s).

Even though no significant difference was indicated, a visual inspection of the temporal profile of pain intensity assessment indicated a higher intensity of pain in the area where cowhage was applied, and treated with vehicle cream for 48 h when compared with all the other experimental conditions (Figure 4B). The itching temporal assessment did not show a significant difference between the time points of application. However, a qualitative decrease is seen in the area treated with *S. ramosissima* cream for 24 h vs. vehicle cream area after 24 h when histamine was applied (Figure 4A).

## 3. Discussion

The current study aimed to investigate the putative analgesic and antipruritic effects of 10% *Salicornia ramosissima* topical application after 24 or 48 h in healthy subjects. 

This study showed that topical application of *S. ramosissima* cream significantly alleviated experimentally provoked itching and increased warmth/heat pain thermal threshold in healthy subjects depending on the time of application of infused cream. 

Effects of halophyte-based creams have not been fully studied on pain and itching sensitivity but literature hint that the plants can have a medical application due to their secondary bioactive metabolites [9]. The aqueous and organic extracts from *Salicornia* spp. are known to include high levels of bioactive compounds among these are alkaloids, fatty acids, lipids, flavonoids, phenolics, quinines, tannins, terpenoids, steroids, saponins, and coumarins [1]. *Salicornia* spp., a part of the halophytes, has been extensively investigated in previous studies, where regulation of proinflammatory cytokines as well as oxidative stress markers, such as nitrogen-oxide production, were proven [5,8,9]. Purified fractions from *S. ramosissima* have been demonstrated to produce a large variety of phytochemicals that contribute to the proposed antioxidant and photo protectant effects [5]. Moreover, a recent study extract of topical *Salicornia* spp. improved the skin surface texture of sun-exposed skin for eight weeks, concluding that the *Salicornia* spp. extract suppresses the misoriented axes of cell division in the basal layer of the skin [12]. Different solvents were used using Soxhlet to find an optimal extraction method for S. ramosissima, including water, ethanol, 40:60 *v/v*% ethanol:water, and ethyl acetate. These solvents have been shown to extract phenolic and antioxidant compounds from *Salicornia brachiata*, these were used in an extract screening experiment [14]. All solvents except for ethyl acetate extracted phenolic and antioxidant compounds. A large amount of bound phenolic compounds was released by acid hydrolysis of the extracts produced. The released phenolic compounds were bound in the lignocellulosic matrix of the lignocellulosic microparticles in the liquid extract. The following membrane filtration with a hydrolyzed and non-hydrolyzed extract showed that the retention of phenolic compounds correlated to the retention of lignocellulose in the membrane. This analysis indicates the need for hydrolysis during the extraction of phenolic compounds, as a larger fraction of the bound phenolics will be liberated. In the present study, the effect of the *S. ramosissima* cream (10% *v:v*) was tested for short periods of 24 or 48 h to evaluate any time-dependent effects. Previous studies evaluated pH, which indicated an alteration of the homeostatic status of the skin [19,20]. TEWL is one of the assessments used to test the homeostasis of the skin barrier by evaluating the amount of water that passively evaporates through the skin to the external environment [13]. Therefore, an intact skin barrier is essential for preventing unjustified evaporation of water in the external environment, which results in low values of TEWL when assessed [13]. Values of TEWL, representing a healthy skin barrier, are averaged around 2–8 g/m^−2/^h^−1^ for the middle forearm and have been shown to increase to 50 or even over 100 g/m^−2/^h^−1^ after exposure to irritants, burns, or severe damage [19,21]. In this present study, a significant increase in TEWL was observed from baseline to post-cream application measurements, highlighting that treatment with *S. ramosissima* cream and vehicle cream induced disruption of the barrier function. This effect could be caused by the composition of the vehicle cream in which the extract has been dissolved. However, a significant reduction in TEWL was found in the group who received the application for 48 h compared with 24 h, indicating a lesser skin barrier alteration 48 h after application. Previous studies have evaluated the effect of plant-based lotion on skin permeability showing how different composition of the lotion and different plant species can modify the TEWL of the skin in physiological and pathological conditions [22,23]. For Salicornia species, only one study has evaluated the skin condition after a long application of *S. europaea,* showing that treatment with *S. europaea* extract helps improve the skin texture, and through a skin model authors showed the importance of the orientation in the stem cells in maintaining skin homeostasis [12].

Moreover, in this study, the effect-induced variation of thermal sensation after Salicornia cream application was evaluated. The transient receptor potential (TRP) family is involved in the transduction of thermal stimuli [24]. For the transduction of heat stimulation ≥43 °C the TRPV1 is activated triggering Aδ- and C-fibers [25,26]. For the perception of warmth (32–39 °C), the TRPV3 channel has been suggested to play a major role [24]. In the present study, a significant decrease in both warmth and heat pain thresholds were found in the Salicornia-treated group as compared with the placebo when 48 h were compared with 24 h of application. This finding highlights the importance of application time, potentially due to the possibility for the extract to reach the dermal-epidermal junction where the receptor is mainly located [21]. In accordance with this statement a previous study has proven the beneficial effects of prolonged application of *S. europea* in healthy subjects only after 8 weeks of repeated applications [12].

In the present study, the antipruritic effect of the *S. ramosissima*, through well-established experimental models of histaminergic and non-histaminergic itch provocations by histamine and cowhage, respectively [27]. Mechanical-evoked itching (MEI) is a typical phenomenon of itch sensitization associated with patients with chronic pruritus such as atopic dermatitis, the phenomenon is also termed hyperkinesis, defined as higher itching intensity induced by a pruritogens stimulus [16,28]. Histamine and cowhage are the two most used models of human itching. The itching can be simulated experimentally in healthy volunteers by the provocation of, e.g., histamine and cowhage. Histamine induces itching by the binding to its receptors (H1-R and H4-R) expressed by a subgroup of mechano-insensitive C-fibers [29,30]. The transmission of histaminergic itch sensation also involves the activation of transient receptor potential vanilloid 1 (TRPV1), expressed on the same subgroup of fibers [31,32], with a consequent influx of Ca^2+^ [29]. On the other side, cowhage binds the protease-activated receptors (PAR2 and PAR4) expressed by a subgroup of polymodal C-fibers. The transmission of cowhage-induced non-histaminergic itching through PmC-fibers is induced also by the downstream activation of transient receptor potential ankyrin 1 (TRPA1) [32]. In the present study, the application of *S. ramosissima* cream for 48 h showed a significant decrease in mechanical itching sensitivity evoked with Von Frey when compared with application at 24 h. No differences were highlighted between the response reported from the area treated with *S. ramosissima* cream and the area treated with vehicle cream. Even though an effect was present after the application of the *S. ramosissima* cream for 48 h, no reduction in hyperkinesis was detected after itch induction with neither histamine nor cowhage. On the contrary, subjects reported an increase in itching intensity in the *S. ramosissima* cream-treated area for 48 h after histamine application compared with 24 h treatment, possibly caused by the increased disruption of the skin barrier. 

Within the cutaneous vasomotor responses, neurogenic inflammation is the most common response to allogenic and purinergic substances [33]. Neurogenic inflammation or flare consists of vasoactive peptide release and hence an increase in superficial blood due to peptidergic sensory nerve fibers activation. Evidence shows the contribution of peripheral nerves in several skin pathologies and that neuromediators are involved in the pathophysiology of, e.g., pruritus and inflammatory responses [33]. In this study, the evaluation of blood flow after histamine application showed a reduction in the area treated for 48 h with *S. ramosissima* cream and vehicle cream, when compared with areas treated after 24 h with no differences between the Salicornia-treated area and the vehicle-cream-treated area. This suggests an unspecific effect of the occlusion but underscores a better effect when used for a prolonged time.

## 4. Materials and Methods

### 4.1. Study Design and Cohort

**Figure 5:**
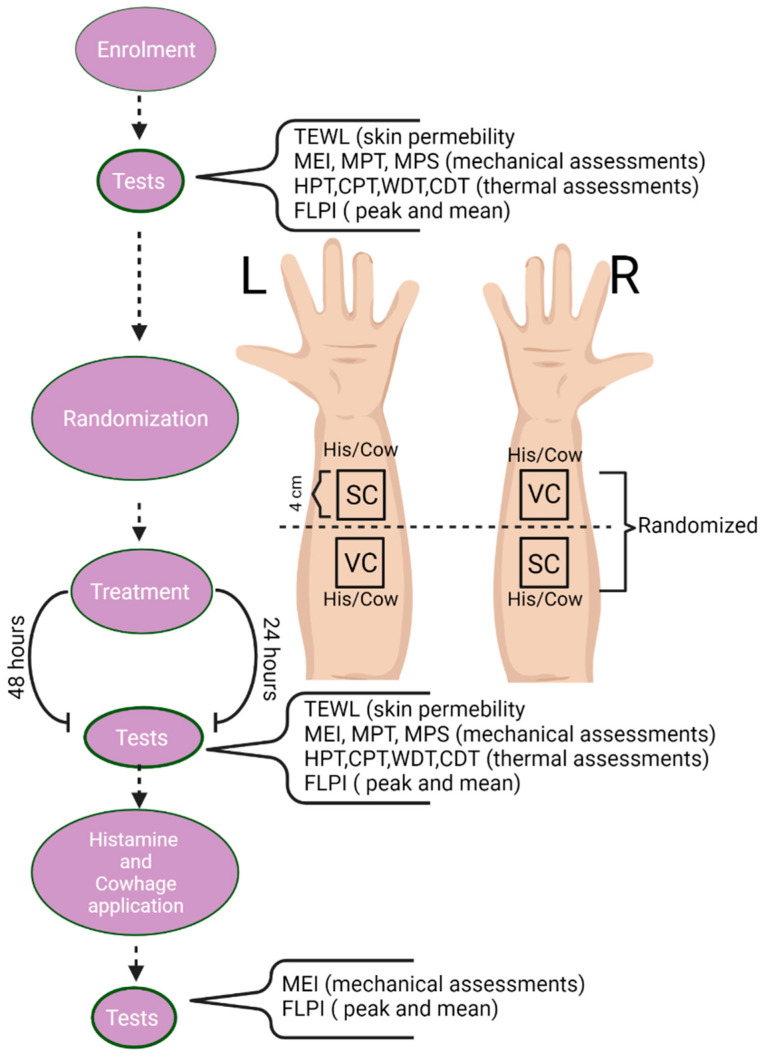
Study design. Flowchart of the study design, sensory testing and vasomotor assessments performed in the 4 areas and randomization of cream application to the volar forearm of healthy participants.

30 participants were recruited (15 males and 15 females, aged 18 to 33, 25.1 ± 2.4 years). Exclusion criteria included pregnancy or lactation, skin disease, use of medications (e.g., painkillers or antihistamine), previous or current neurologic, musculoskeletal, or mental illnesses, acute or chronic itching or pain. Following the Helsinki Declaration, all subjects signed an informed consent form, and the Regional Ethics Committee approved the protocol (N-20200072). The protocol was registered on clinicaltrials.gov (accessed on 20 December 2021) (NCT04635254). The overall study was designed as a randomized, single-blinded, controlled trial that included two sessions, which lasted over two days. The subjects were divided into two groups, with 15 subjects in the “24 h” group and 15 subjects in the “48 h” group, depending on their participation in the second session after 24 or 48 h. The volar forearms of each subject were divided into two squared areas (4 × 4 cm), located 4 cm apart. During the first session, quantitative sensory tests and measurement of neurogenic flare were performed on the designated areas to obtain baseline data. After the tests, one area on each arm was treated with *S. ramosissima* cream (SC) (10% Salicornia extract), and the other area was treated with a vehicle cream (VC) (similar in composition to the *S. ramosissima* cream but with the absence of active plant extract). The placement of SC and VC on each arm was randomized. The second session was either performed after 24 or 48 h and included, after removing the cream from the treated areas, the identical tests ran during the 1st session. At the end of this last session, histamine and cowhage were applied to the designated areas. At 10 min after applying pruritogens, tests were run (Figure 1). The histamine and cowhage applications were randomized so that each arm received only histamine or cowhage (Figure 5).

### 4.2. Extraction of Salicornia Ramosissima

To obtain extract used from *S. ramosissima* for production of cream, 2.00 kg shredded *Salicornia ramosissima* was extracted using the Soxhlet extraction method in 25 L water. The *Salicornia ramosissima* was shredded to 8 mm, and particles smaller than 2 mm were sifted off to avoid equipment clogging. The extraction was run for 8 h with a Soxhlet cycle time of 45 min. After extraction, the extract was siphoned into jerry cans, and frozen until used.

### 4.3. Cream Composition, Itch, and Pain Induction Parameters

Salicornia cream 10% *v:v*: In two 4 × 4 cm squared areas on the forearms, 2 g of cream (containing 10% of *Salicornia ramosissima* extract, DK Beauty, Brande, Denmark) were applied under topical occlusion (TegaDerm) to facilitate absorption for either 24 or 48 h. At the end of the 24 or 48 h applications, before sensory tests, the excess cream applied was removed. The same approach was used for vehicle cream (DK Beauty, Brande, Denmark), used as a placebo. 

Histamine: In session 2, histamine (1% solution) was delivered by using standard allergy skin prick test (SPT) lancets. Through the lancets (with 1 mm shouldered tip), a small amount of test substance was introduced extremely locally and approximately at the dermo-epidermal junction [29,30]. In this study, a weight-controlled SPT (120 g) was used to decrease the variability of the application method. At 24 or 48 h after cream application, a small drop of histamine dihydrochloride (1%, in saline) was applied to the center of the designated areas (one pretreated with Salicornia cream and one with vehicle cream) on the volar forearm, followed by a lancet prick through the drop.

Cowhage: In session 2, cowhage spicules were counted under a stereo microscope, and 25–30 spicules were picked with negative action tweezers and were gently rubbed through 1 cm diameter skin areas (one pretreated with Salicornia cream and one with vehicle cream). Application of spicules breaches the keratinous layer of the skin (0.05–0.15 mm) without reaching the proximity of circulation [34,35]. The active compound (mucunain) has previously been shown to be released in the nanogram range [36]. After measurements, the spicules were rapidly removed using tape (gently applying it and stripping it off the skin a few times).

#### 4.3.1. Microvascular Reactivity

Full-field laser perfusion imaging (FLPI, Moor Instruments, Axminster, Devon, UK) was used to evaluate cutaneous neurogenic inflammation (quantified by superficial blood perfusion) at baseline, 24 or 48 h after cream application, and after histamine and cowhage application. Pictures were taken with the device placed 25 cm above the skin ar-ea. A 5 Hz display rate, 8.3 ms exposure time, and 160 units of gain were used. The FLPI data were extracted using a region of interest (ROI) approach, and mean and peak perfusion values were obtained. 

#### 4.3.2. Evaluation of Skin Permeability

To assess variance in skin permeability, trans-epidermal water loss was measured using DermaLab Single (Cortex Technology, Hadsund, Denmark) to allow the evaluation of changes in the humidity of the skin. This was carried out by measuring the areas on the designated volar forearms using a probe cylinder with humidity sensors using Dermalab SkinLab 1.03 software, at baseline and after cream application.

#### 4.3.3. Measurement of Mechanically Evoked Itch

To assess a mechanically evoked itch, three von Frey filaments were used: 4.08, 4.16 and 4.31 (1.0, 1.4, 2.0 g, respectively; North Coast Medical, Gilroy, CA, USA). The assessment consisted of three pricks repeated three times in succession with each filament. After each stimulation (nine in total), participants were instructed to report the itching induced on a numerical rating scale (NRS) from 0 to 10 (0 = “no itch”; 10 = “worst imaginable itch”). The total average of assessments was calculated. This method has previously been described [16].

#### 4.3.4. Measurement of Mechanical Pain Thresholds (MPT) and Mechanical Pain Sensitivity (MPS) 

To assess the MPT, a pin-prick set (MRC Systems GmbH, Germany) was used. The set contains eight needles having the same diameter of 0.6 mm at the tip, and different force applications ranging from 8 to 512 mN. Starting from the lightest pin, and at a rate of 2 s on, 2 s off in ascending order, each stimulator was applied perpendicularly at the center of the area until the subject reported a perception of sharpness or pain (pin-prick pain). Through this method, known as the “method of limits”, five thresholds were obtained using a series of ascending or descending stimuli, and the final threshold was obtained by calculating the geometric mean. 

The same pin-prick set was also used to assess the MPS. Starting with the lightest pin, each stimulator was applied using an ascending order perpendicularly at the center of the area, and the subject was asked to rate the pain perceived after each stimulation on an NRS ranging from 0–10 (0 = “no pain”; 10 = “worst imaginable pain”). This assessment was run in duplicate.

#### 4.3.5. Thermal Sensitivity 

Cold detection threshold (CDT), warm detection threshold (WDT), cold pain threshold (CPT) and heat pain threshold (HPT) was measured using a thermal stimulator Medoc Pathway (Medoc Ltd., Ramat Yishay, Israel). A thermal stimulator probe (3 × 3 cm) with a starting temperature of 32 °C was placed on each application area. An ascending or descending ramp stimulus (1 °C/s) was delivered until the subject identified the relevant threshold and pressed the button on a mouse to stop the measurement. For WDT or CDT the relevant threshold was the perception of a slight change in the temperature feeling (warm, or cold sensation); for CPT and HPT the detection was intended as a painful sensation. After the click, the temperature returned to the baseline at a rate of 5 °C/s. For safety, a cut-off temperature of 52 °C was used. The results were calculated as the arithmetic mean of the thresholds obtained from three repeated ramps.

The same thermal stimulator used above was used to measure Supra-threshold heat sensitivity (STHS). For this measurement, the participant was asked to rate the pain (from 0 to 10) to two supra-threshold heat pain stimuli. Each stimulation started and ended with a temperature of 32 °C, using an increasing ramp of 5 °C/s, and a plateau of 3 s during which the temperature reached 50 °C before decreasing at a speed of 5 °C/s. The test was performed on the treated/placebo areas. The result was the average of the two values obtained.

#### 4.3.6. Assessment of Itching and Pain Intensities

To assess the itching and pain intensities and durations after pruritogens application, two computerized 100 mm VASs (eVAS Software V 1.0, Aalborg University, Aalborg, Denmark) were installed on a Samsung Note 10.1 Tablet (Samsung, Seoul, South Korea were used (one for itching and one for pain ratings). The two VAS scales were ranging from 0 to 100, whit 0 indicating “no itch” or “no pain” and 100 indicating the “worst imaginable itch” or “worst imaginable pain”. Each participant had to rate the itching continuously for 9 min and the pain felt following histamine and cowhage application. Pain and itching were sampled at 0.2 Hz; thus, a value could be extracted every 5 s. 

#### 4.3.7. Statistical Analysis 

The SPSS (v26, IBM Corporation, 10504-1722Armonk, New York, NY, USA) software was used to perform statistical analysis. Data from all assessments were tested for normality using the Shapiro–Wilk normality test. Data were analyzed using the repeated measure analysis of variance (RM- ANOVAs) followed by a Bonferroni post hoc test, and a *t*-test for differences in effects between 24 and 48 h treatment. A significance value of *p* ≤ 0.05 was considered statistically significant. Histamine- and cowhage-induced temporal itching profiles were generated and the area under the curve (AUC) and the peak itching intensities were extracted. RM-ANOVAs were constructed using the following factors: treatment (vehicle/*Salicornia*), time (baseline, first day and post-pruritogens), pruritogen (histamine/cowhage). For data non-normally distributed, the Friedman test was run as a non-parametric equivalent of RM-ANOVA. To analyze differences between applications at 24 and 48 h, *t*-tests were performed. Graph plotting was realized in GraphPad Prism 6 (GraphPad Software Inc., 92108 San Diego, CA, USA).

## 5. Conclusions

Thus, in this study, the effects of *Salicornia ramosissima*-infused cream were investigated in healthy groups treated after 24 or 48 h. The obtained data indicate an overall effect of the bioactive cream to reduce mechanically evoked itching, induce an analgesic thermal effect and regulate the skin barrier architecture. However, the study design and time frame selected also necessitate the need for further assessment of the long-term effect after prolonged use. The current interest in nutraceuticals and renewable bioactive compounds for the pharmaceutical industry is rising due to the nutritional potential, safety, and therapeutic effect, as well as on the grounds of increased consumer demand. Future use of green technologies and renewable ingredients, such as the *S. ramosissima*-infused skin cream, as a putative primary treatment to reduce symptoms such as itching and pain in different skin diseases, with psoriasis and atopic dermatitis among them.

## Figures and Tables

**Table 1:** Demographic Data. A total of 30 human subjects were included in the study, 15 males and 15 females. The average age is reported as mean ± standard deviation (SD). All of the included subjects completed the trial.

Demographics	
Participants (*n*)	30
Female (%)	15 (50%)
Male (%)	15 (50%)
Age (mean ± SD)	25.1 ± 2.46

## Data Availability

The data presented in this study are available in article.
